# Weak Tradeoff and Strong Segmentation Among Plant Hydraulic Traits During Seasonal Variation in Four Woody Species

**DOI:** 10.3389/fpls.2020.585674

**Published:** 2020-11-24

**Authors:** Xiao Liu, Qiang Li, Feng Wang, Xiaohan Sun, Ning Wang, Huijia Song, Rong Cui, Pan Wu, Ning Du, Hui Wang, Renqing Wang

**Affiliations:** ^1^School of Life Sciences, Institute of Ecology and Biodiversity, Shandong University, Qingdao, China; ^2^Shandong Provincial Engineering and Technology Research Center for Vegetation Ecology, Shandong University, Qingdao, China; ^3^Qingdao Forest Ecology Research Station of National Forestry and Grassland Administration, Shandong University, Qingdao, China

**Keywords:** annual, embolism resistance, habitat partitioning, niche divergence, warm temperate zone, water relation

## Abstract

Plants may maintain long-term xylem function via efficiency-safety tradeoff and segmentation. Most studies focus on the growing season and community level. We studied species with different efficiency-safety tradeoff strategies, *Quercus acutissima*, *Robinia pseudoacacia*, *Vitex negundo* var. *heterophylla*, and *Rhus typhina*, to determine the seasonality of this mechanism. We separated their branches into perennial shoots and terminal twigs and monitored their midday water potential (Ψ_md_), relative water content (RWC), stem-specific hydraulic conductivity (K_s_), loss of 12, 50, and 88% of maximum efficiency (i.e., P_12_, P_50_, P_88_) for 2 years. There were no correlations between water relations (Ψ_md_, RWC, K_s_) and embolism resistance traits (P_12_, P_50_, P_88_) but they significantly differed between the perennial shoots and terminal twigs. All species had weak annual hydraulic efficiency-safety tradeoff but strong segmentation between the perennial shoots and the terminal twigs. *R. pseudoacacia* used a high-efficiency, low-safety strategy, whereas *R. typhina* used a high-safety, low-efficiency strategy. *Q. acutissima* and *V. negundo* var. *heterophylla* alternated these strategies. This mechanism provides a potential basis for habitat partitioning and niche divergence in the changing warm temperate zone environment.

## Introduction

Precipitation patterns have been substantially altered as consequence of global climate change (Easterling et al., 2000; Högy et al., 2013; Gimbel et al., 2015; Ge et al., 2017). This phenomenon has aggravated the existing uneven seasonal water distribution (and, by extension, soil, and air moisture status) in the warm temperate zone (Luan et al., 2011; Corlett, 2016). Uneven water distribution leads to variable water availability, which strongly influences plant performance and forest functional composition by affecting hydraulic traits (Ni, 2003; Del Grosso et al., 2008; Allen et al., 2010; Esquivel-Muelbert et al., 2017), which, in turn, determine the ability of the plant to adapt to seasonality and climate change.

Xylem traits regulate plant hydraulic functioning and play an important role in plant survival and growth (Choat et al., 2018). To ensure plant health, the xylem must remain free of embolisms under both favorable and water stress conditions (Charrier et al., 2018; Varela et al., 2018; Zhang et al., 2018). Xylem-dependent hydraulic traits including water relations and embolism resistance traits are crucial for plant growth and adaptation.

The hydraulic efficiency-safety tradeoff is a balance (i.e., negative correlation) among the various traits (Grossiord et al., 2020). The efficiency-safety tradeoff often occurs in different tissues and structures, changes with the season and plant growth (Holmlund et al., 2016; Prendin et al., 2018), maintains xylem function (Aguilar-Romero et al., 2017; van der Sande et al., 2019). The efficiency-safety tradeoff has been proved in hydraulic conductivity, which is comparatively higher during the growing season (Magnani and Borghetti, 1995; Jaquish and Ewers, 2001; Aranda et al., 2005), while the plant embolism resistance capacity exhibits the opposite trend (Varela et al., 2018; Zhang et al., 2018; but see Gleason et al., 2016). The efficiency-safety tradeoff is driven by environmental factors and contributes to habitat partitioning and niche divergence in sympatric species (Aguilar-Romero et al., 2017; Santiago et al., 2018; Rosas et al., 2019). The efficiency-safety tradeoff combines the hydraulic traits at the xylem level (Aguilar-Romero et al., 2017; van der Sande et al., 2019). Prior studies have focused on growing-season hydraulic traits. Nevertheless, few studies have investigated the hydraulic efficiency-safety tradeoff for the whole year. In the non-growing season after leaves abscise, the rest of plant organs (e.g., branch and root) still need water transport and there may be an efficiency-safety tradeoff. Evidently, it was not enough to reflect plant strategies only sampling and measuring during the growing season. Therefore, what are the efficiency-safety tradeoffs among plant tissue traits throughout the year?

Segmentation is another important mechanism to keep plant normal function driven by variations of hydraulic traits in different plant tissues and organs (Wason et al., 2018). Firstly, the hydraulic segmentation hypothesis (HSH) predicts a “hydraulic constriction” at the organs’ junction, driving the leaves, terminal twigs, and fine roots to be more hydraulically resistant (or less conductive). This differentiation of hydraulic resistance may drive a larger, steeper water potential gradient and hydraulic conductivity difference between tissues or organs (Levionnois et al., 2020). This mechanism enables plants to have different water relations among organs, to make sure that xylem in distal organs with high energetic costs have better water conditions for plant growth and adaptation. Then, the hydraulic vulnerability segmentation hypothesis (HVSH) predicts that the xylem in distal organs with low energetic costs (leaves, terminal twigs, and fine roots) are more prone to embolism than the xylem in organs with higher energetic costs (perennial shoots, trunk, and main root) (Jin et al., 2018). This mechanism enables drought-stressed plants to sacrifice highly vulnerable plant segments by confining embolism to the distal sectors, keeping the remaining parts hydraulically active, and protecting the central parts of the water transport system (Losso et al., 2019). Numerous studies have focused on growing-season segmentation in distal plant organs including the leaves, branches, and roots. The time to desiccation is longest in species with strong embolism resistance and vulnerability segmentation which enable plants to adapt to complex environments (Johnson et al., 2016; Blackman et al., 2019; Losso et al., 2019; Skelton et al., 2019). Liu et al. (2015) examined segmentation between leaflet laminae and compound leaf petioles in two compound leaf tree species during the growing season. They demonstrated that under current climate conditions, the main segmentation may be located between the leaflet laminae and the compound leaf petioles. However, little is known regarding seasonal changes in segmentation. Unlike the branches, the leaves are not permanent all year in warm temperate deciduous broadleaf forests. Thus, we must pay attention to segmentation seasonality between perennial shoots and terminal twigs to establish whether segmentation is annually permanent.

Species use various strategies to adapt to seasonal environments. Charrier et al. (2018) reported that grapevine (*Vitis vinifera* L.) stem hydraulics changed seasonally to endure unstable hydrothermal conditions. However, embolism resistance in *Acer mono* Maxim. was reported to be seasonally constant, nevertheless, seasonal changes were detected in the mechanical traits of the pit membranes (Zhang et al., 2018). Petruzzellis et al. (2018) showed that alien species tended to sacrifice safety for efficiency more than native species. The adoption of diverse strategies by different species may lead to niche differentiation (Aguilar-Romero et al., 2017; Petruzzellis et al., 2018; Santiago et al., 2018). Seasonal strategies among sympatric species have seldom been considered especially concerning the hydraulic efficiency-safety tradeoff and segmentation. The warm temperate zone is characterized by four distinct seasons widely varying in hydrothermal conditions. Consequently, this type of environment provides ample opportunity to study seasonal changes in hydraulic efficiency-safety tradeoff and segmentation.

Four common woody plants were selected for the present study. *Quercus acutissima* Carr. and *Robinia pseudoacacia* L. are dominant species in the tree layer of temperate deciduous broadleaved forests in Northern China. *Vitex negundo* L. var. *heterophylla* (Franch.) Rehd. predominates in the temperate deciduous broadleaved shrub layer, while *Rhus typhina* L. is a competitive alien species in the forests. They are widely dispersed sympatric tree species in Northern China (Wang and Zhou, 2000; Fang et al., 2011), and in recent research, they are renowned for their drought tolerance and restorative capacity in the warm temperate zone (Xu et al., 2009; Du et al., 2017; Li et al., 2019). According to a previous study, *Q. acutissima* and *V. negundo* var. *heterophylla* were classified as high-safety species while *R. pseudoacacia* was categorized as a high-efficiency species (Moser et al., 2016; Li et al., 2019). To the best of our knowledge, no prior studies have examined or reported on efficiency-safety tradeoff and segmentation in *R*. *typhina*. Here, we set up an ordinary garden experiment to elucidate seasonal changes in hydraulic efficiency-safety tradeoff and segmentation and measured the traits of perennial shoots and terminal twigs. Measurements included midday water potential (Ψ_md_), relative water content (RWC), stem-specific hydraulic conductivity (K_s_). We investigated a range of safety definitions, including the loss of 12, 50, and 88% of maximum efficiency (i.e., P_12_, P_50_, P_88_). P_12_ is the air-entry point, it is an estimate of the xylem tension at which pit membranes are overcome within the conducting xylem and when cavitation starts (Domec and Gartner, 2001; Torres-Ruiz et al., 2017); P_50_ is the fastest drop point, it is described as the steepest part of the vulnerability curve (Choat et al., 2012; Gleason et al., 2016); P_88_ is the upper inflection point, it likely represents a lethal point and appears to be the value that reflects the inherent risk to critical hydraulic failure for most angiosperm (Choat et al., 2012; Scholz et al., 2014). These functional traits capture important hydraulic trait axes such as water relations [Ψ_md_, RWC, hydraulic efficiency (K_s_)]; and embolism resistance (hydraulic safety; P_12_, P_50_, P_88_). We hypothesized that for the whole year, (1) hydraulic efficiency-safety tradeoff exists in all the four species, (2) segmentation among various tissues always occurs in the two tree species while it may only occur during the dry period in the two shrub species, and (3) different species may implement various strategies to adapt to changing environments, and tree species may adopt safer tradeoff and more segmentation than shrub species.

## Materials and Methods

### Study Site

The study was conducted at the Fanggan Research Station of Shandong University in Jinan, Shandong Province, China (36° 26′ N, 117° 27′ E). The region has a warm temperate monsoon climate with an average annual temperature of 13 ± 1°C and annual precipitation of 700 ± 100 mm. The soil type is yellow cinnamon and the parent material is limestone (Li et al., 2018, 2019). The experiment was conducted in the common garden at the research station. Temperature (T,°C) and relative humidity (RH) were measured every 10 min with a HOBO datalogger (U12-012; Onset Computer Corporation, Bourne, MA, United States). The monthly average precipitation (MAP) was acquired from the nearby Xueye Meteorological Station located ∼1 km from the Fanggan Research Station.

### Plant Materials

The study was run from July 2016 to June 2018. Seeds of *Quercus acutissima* Carr., *Robinia pseudoacacia* L., *Vitex negundo* L. var. *heterophylla* (Franch.) Rehd., and *Rhus typhina* L. were collected from the mountain near the research station in October 2006. In 2007, the seeds were planted and irrigated in the common garden at the station. By the time the study began in 2016, all plant materials were ∼10 y. Fifteen healthy individuals of similar size were selected from each species for plant hydraulic traits measurements, and their height and diameter were measured (Supplementary Table S1).

### Experimental Design

A well-illuminated, intact, healthy branch on each individual was cut into perennial shoots (PS) and terminal twigs (TT). Generally, TT had green bark and a small diameter (less than 4 mm) while PS often had brown bark and a large diameter (more than 4 mm). TT and PS were used for the plant hydraulic traits determinations. Firstly, 15 individuals per species were selected for plant predawn (2 h before sunrise) water potential (Ψ_pd_), midday (2 h at noon) water potential (Ψ_md_), predawn relative water content (RWC), and predawn stem-specific hydraulic conductivity (K_s_) measurements after >2 consecutive sunny days in the middle of each month from July 2016 to June 2018. Meanwhile, 6 of 15 individuals per species were randomly selected and used to determine hydraulic vulnerability curves in the middle of January, March, May, July, September, and November 2017.

### Water Potential

Water potential was measured *in vitro* in a pressure chamber (1505D-EXP; PMS Instrument Company, Albany, OR, United States). Samples for the predawn and midday water potentials (Ψ_md_, MPa) were taken before sunrise (2 h between 02:00–06:00) and at midday (2 h between 11:00–15:00), respectively. Predawn water potential was best defined as a measure of soil water availability at predawn (Pérez-Harguindeguy et al., 2016), thus, predawn water potential was used as an estimation of soil water potential (Ψ_s_, MPa) in our study. Samples were cut from the trees, sealed in plastic bags containing moist paper towels, and stored in a cooler before the water potentials were measured in a laboratory near the common garden. To maximize measurement accuracy, only a few branches were tested at one time. The other branches were not excised until those already harvested were measured. The samples were not removed from their paper towel wrapping and all water potentials were measured in the laboratory within 30 min after excision.

### Relative Water Content

After the water potential measurement, the leaves were removed from the stems and the fresh weight of the stripped stems (W_f_, g) was measured. The segments were stored in water in plastic bags for 24 h and the stem saturated weight (W_s_, g) was measured. The samples were placed in an oven at 80°C for 48 h and the dry weight (W_*d*_, g) was measured. The stem relative water content (RWC) was calculated as follows:

(1) RWC=(W-fW)d/(W-sW)d,

### Stem-Specific Hydraulic Conductivities

The hydraulic conductivity measurement was taken at the same time as the water potential measurement. Branches ≥ 50 cm long were excised and ∼5 cm of each stem end was cut off underwater. The submerged stems were then transported promptly to the laboratory and the crowns were covered with black plastic bags. The leaves were removed and ∼5 cm of each stem end was recut underwater, then, branches were separated into terminal twigs (TT) and perennial shoots (PS). Each segment was ∼15 cm long. The segments were connected to a hydraulic conductivity measurement system containing degassed, filtered 20.0 mM KCl solution. A 40-cm hydraulic head generated hydrostatic pressure to impel water through the segments. The hydraulic conductivity (K_h_, kg m s^–1^ MPa^–1^) was calculated as follows:

(2) $${\text{K}}_{\text{h}}=\frac{{\text{LQ}}_{\text{m}}}{\text{p}}$$

where L is the stem length (m), Q_m_ is the mass of water through a stem per unit time (kg s^–1^), and p is the water pressure across the segment (MPa). The stem-specific hydraulic conductivity (K_s_, kg m^–1^ s^–1^ MPa^–1^) was calculated as the ratio of K_h_ to the stem total cross-sectional area.

### Xylem Vulnerability Curves

The vulnerability of stem xylem to cavitation was determined by the air-injection method. Samples were collected after K_s_ measurement in January, March, May, July, September, and November 2017. The sampling method was the same as that of K_s_. The maximum hydraulic conductivity (K_m_, kg m^–1^ s^–1^ MPa^–1^) was measured after the segments were flushed for 20 min with degassed 20 mM KCl solution under 0.10 MPa pressure to remove any air bubbles in the xylem (Pammenter and Van der Willigen, 1998; Sperry et al., 2008; Pérez-Harguindeguy et al., 2016). The segments were then placed in double-sleeved air-injection chambers (1505D-EXP; PMS Instrument Company, Albany, OR, United States). K_h_ was measured after the segments were exposed to increasing pressure ranging from 0 to 10 MPa at 0.5 MPa increments. To plot the curve accurately, a 0.1 MPa pressure increment was used when the percentage loss of hydraulic conductivity (PLC) was >20% and was maintained until the PLC was >70%. Using a regulator, the pressure was held constant for 5 min at each level. After the pressure was released, the injected samples were allowed to equilibrate for ∼10 min until no bubbles emerged from the xylem. At that point, the post-injection K_h_ was determined. The post-injection PLC at each pressure level was calculated as follows:

(3) $$\text{PLC}=\left({\text{K}}_{\text{m}}-{\text{K}}_{\text{h}}\right)/{\text{K}}_{\text{m}}\times 100\%$$

The hydraulic vulnerability curves were fitted with a Weibull cumulative distribution function (CDF) as follows:

$$\mathrm{PLC}=f\left(P;A,B\right)=1-\exp\left[-{\left(\frac{\text{P}}{\text{A}}\right)}^{\text{B}}\right]$$

where P (MPa) is the progressively increased air-injection pressure and A and B are constants matching the Weibull CDF, then the embolism resistance traits, P_12_, P_50_, P_88_ were calculated as the pressure at 12, 50, and 88% PLC, respectively.

### Statistics

Compared with RH and Ψ_s_, the effect of MAP on plant hydraulic traits is indirect, thus, we chose T, RH, Ψ_s_ to quantify different seasons and months in statistics. The data were first tested for normality and homogeneity. A Spearman correlation analysis was conducted to analyze the associations among T, RH, Ψ_s_, Ψ_md_, RWC, K_s_, P_12_, P_50_, and P_88_. A one-tailed paired *t*-test was used to identify the differences between PS and TT concerning all plant hydraulic traits. We also calculated the standard deviation (SD) of water relations using the monthly average data. Statistical analyses were performed in SPSS v. 25 (IBM Corp., Armonk, NY, United States), the critical α-value was set at 0.05. P, K_m_, and K_h_ at different P were used to fit a Weibull CDF. Curve-fitting and embolism resistance traits calculations were conducted in MATLAB v. 2016a (MathWorks Inc., Natick, MA, United States), and all the co-efficient of determination, R^2^, were more than 0.99. All graphs were plotted in Origin v. 2019b (OriginLab Co., Northampton, MA, United States).

## Results

**FIGURE 1:**
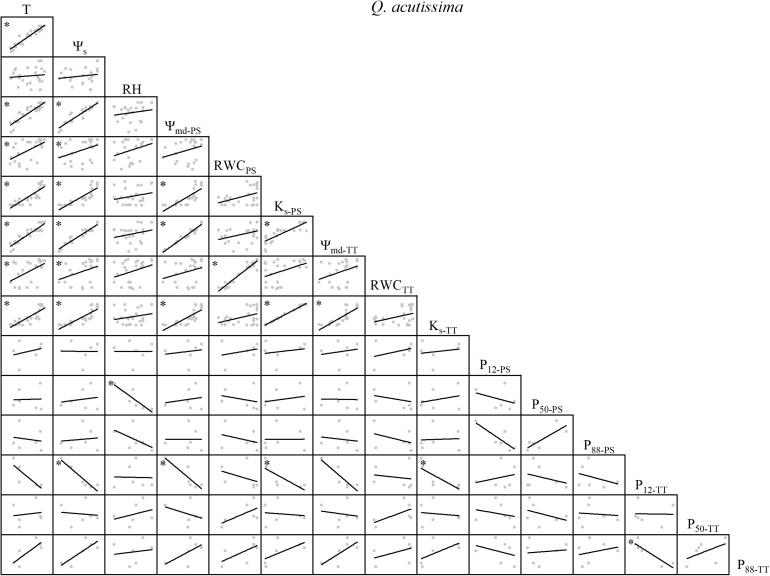
Spearman correlation analysis between environmental factors and plant hydraulic traits in *Q. acutissima*. Data are the mean of each month, *n* = 24 in T, Ψ_s_, RH, Ψ_md–PS_, RWC_PS_, K_s–PS_, Ψ_md–TT_, RWC_TT_, and K_s–TT_; *n* = 6 in P_12–PS_, P_50–PS_, P_88–PS_, P_12–TT_, P_50–TT_, and P_88–TT_. Lines represent the relevant trend. T, air temperature, °C; Ψ_s_, soil water potential, MPa; RH, relative humidity, %; Ψ_md–PS_, midday water potential of perennial shoots, MPa; RWC_PS_, relative water content of perennial shoots; K_s–PS_, stem-specific hydraulic conductivity of perennial shoots, kg m^–1^ s^–1^ MPa^–1^; P_12–PS_, air-entry point of perennial shoots, MPa; P_50–PS_, fastest drop point of perennial shoots, MPa; P_88–PS_, upper inflection point of perennial shoots, MPa; Ψ_md–TT_, midday water potential of terminal twigs, MPa; RWC_TT_, relative water content of terminal twigs; K_s–TT_, stem-specific hydraulic conductivity of terminal twigs, kg m^–1^ s^–1^ MPa^–1^; P_12–TT_, air-entry point of terminal twigs, MPa; P_50–TT_, fastest drop point of terminal twigs, MPa; P_88–TT_, upper inflection point of terminal twigs, MPa. *P < 0.05.

**FIGURE 2:**
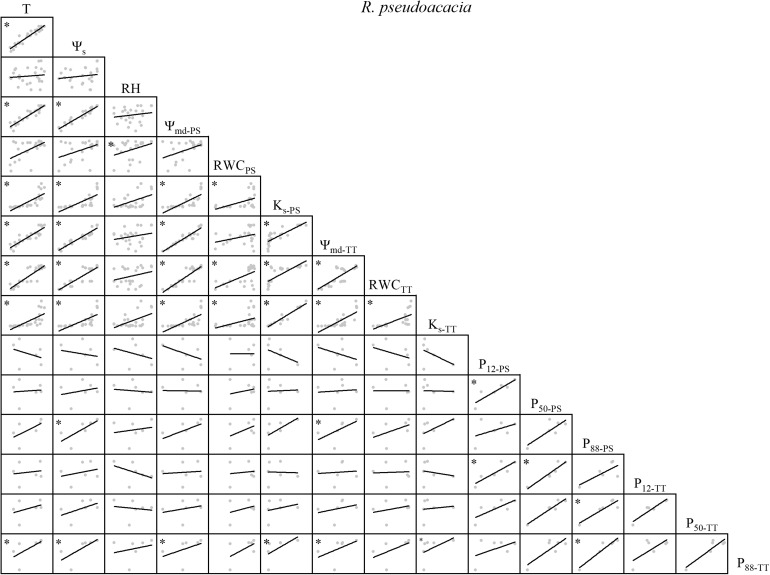
Spearman correlation analysis between environmental factors and plant hydraulic traits in *R. pseudoacacia*. Data are the mean of each month, *n* = 24 in T, Ψ_s_, RH, Ψ_md–PS_, RWC_PS_, K_s–PS_, Ψ_md–TT_, RWC_TT_, and K_s–TT_; *n* = 6 in P_12–PS_, P_50–PS_, P_88–PS_, P_12–TT_, P_50–TT_, and P_88–TT_. Lines represent the relevant trend. T, air temperature, °C; Ψ_s_, soil water potential, MPa; RH, relative humidity,%; Ψ_md–PS_, midday water potential of perennial shoots, MPa; RWC_PS_, relative water content of perennial shoots; K_s–PS_, stem-specific hydraulic conductivity of perennial shoots, kg m^–1^ s^–1^ MPa^–1^; P_12–PS_, air-entry point of perennial shoots, MPa; P_50–PS_, fastest drop point of perennial shoots, MPa; P_88–PS_, upper inflection point of perennial shoots, MPa; Ψ_md–TT_, midday water potential of terminal twigs, MPa; RWC_TT_, relative water content of terminal twigs; K_s–TT_, stem-specific hydraulic conductivity of terminal twigs, kg m^–1^ s^–1^ MPa^–1^; P_12–TT_, air-entry point of terminal twigs, MPa; P_50–TT_, fastest drop point of terminal twigs, MPa; P_88–TT_, upper inflection point of terminal twigs, MPa. *P < 0.05.

**FIGURE 3:**
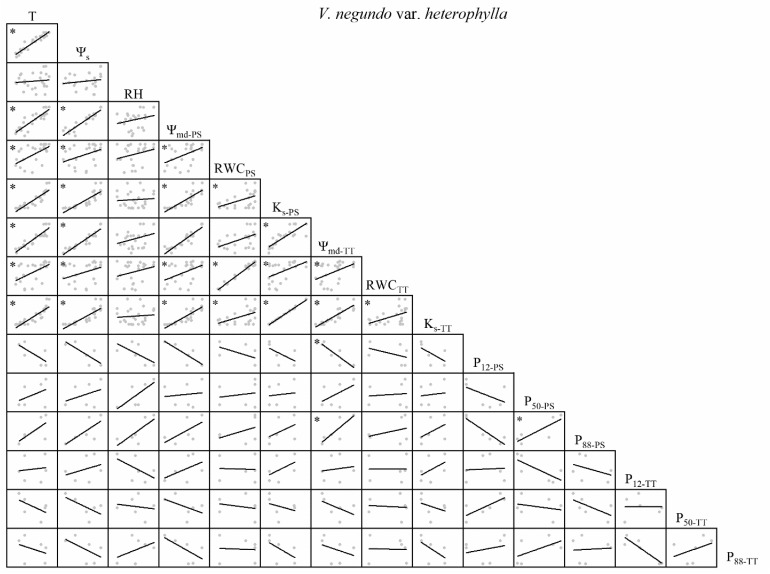
Spearman correlation analysis between environmental factors and plant hydraulic traits in *V. negundo* var. *heterophylla*. Data are the mean of each month, *n* = 24 in T, Ψ_s_, RH, Ψ_md–PS_, RWC_PS_, K_s–PS_, Ψ_md–TT_, RWC_TT_, and K_s–TT_; *n* = 6 in P_12–PS_, P_50–PS_, P_88–PS_, P_12–TT_, P_50–TT_, and P_88–TT_. Lines represent the relevant trend. T, air temperature, °C; Ψ_s_, soil water potential, MPa; RH, relative humidity,%; Ψ_md–PS_, midday water potential of perennial shoots, MPa; RWC_PS_, relative water content of perennial shoots; K_s–PS_, stem-specific hydraulic conductivity of perennial shoots, kg m^–1^ s^–1^ MPa^–1^; P_12–PS_, air-entry point of perennial shoots, MPa; P_50–PS_, fastest drop point of perennial shoots, MPa; P_88–PS_, upper inflection point of perennial shoots, MPa; Ψ_md–TT_, midday water potential of terminal twigs, MPa; RWC_TT_, relative water content of terminal twigs; K_s–TT_, stem-specific hydraulic conductivity of terminal twigs, kg m^–1^ s^–1^ MPa^–1^; P_12–TT_, air-entry point of terminal twigs, MPa; P_50–TT_, fastest drop point of terminal twigs, MPa; P_88–TT_, upper inflection point of terminal twigs, MPa. *P < 0.05.

**FIGURE 4:**
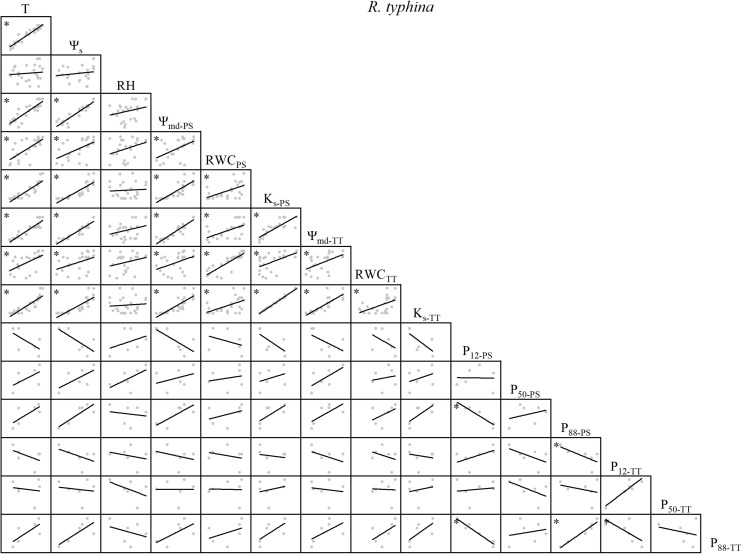
Spearman correlation analysis between environmental factors and plant hydraulic traits in *R. typhina*. Data are the mean of each month, *n* = 24 in T, Ψ_s_, RH, Ψ_md–PS_, RWC_PS_, K_s–PS_, Ψ_md–TT_, RWC_TT_, and K_s–TT_; *n* = 6 in P_12–PS_, P_50–PS_, P_88–PS_, P_12–TT_, P_50–TT_, and P_88–TT_. Lines represent the relevant trend. T, air temperature, °C; Ψ_s_, soil water potential, MPa; RH, relative humidity,%; Ψ_md–PS_, midday water potential of perennial shoots, MPa; RWC_PS_, relative water content of perennial shoots; K_s–PS_, stem-specific hydraulic conductivity of perennial shoots, kg m^–1^ s^–1^ MPa^–1^; P_12–PS_, air-entry point of perennial shoots, MPa; P_50–PS_, fastest drop point of perennial shoots, MPa; P_88–PS_, upper inflection point of perennial shoots, MPa; Ψ_md–TT_, midday water potential of terminal twigs, MPa; RWC_TT_, relative water content of terminal twigs; K_s–TT_, stem-specific hydraulic conductivity of terminal twigs, kg m^–1^ s^–1^ MPa^–1^; P_12–TT_, air-entry point of terminal twigs, MPa; P_50–TT_, fastest drop point of terminal twigs, MPa; P_88–TT_, upper inflection point of terminal twigs, MPa. *P < 0.05.

There were variable correlations among the environmental factors, water relations, and embolism resistance traits. T and Ψ_s_ were significantly positively correlated but neither were correlated with RH (Figures 1–4). The observed changes in water relations of all species were consistent with the alterations in T and Ψ_s_ and were significantly positively correlated (Figures 1–4). There was a non-significant positive correlation between RWC_PS_ and T or Ψ_s_ for *R*. *pseudoacacia*. However, RWC_PS_ was significantly positively correlated with RH (Figure 2).

The correlations among water relations were positive for *Q. acutissima* but only the associations between Ψ_md_ and K_s_ were significant for this species and between PS and TT. P_12–PS_, P_50–TT_, and P_88–TT_ were positively correlated with T, Ψ_s_, and water relations. In contrast, the other embolism resistance traits were negatively correlated with T, Ψ_s_, and water relations (Figure 1).

Overall, there were significant positive correlations among water relations for *R. pseudoacacia*. In general, P_88–PS_, P_50–TT_, and P_88–TT_ were positively correlated with T, Ψ_s_, and water relations whereas most of the other embolism resistance traits were negatively correlated with T, Ψ_s_, and water relations (Figure 2).

For *V. negundo* var. *heterophylla*, water relations were significantly positively correlated. P_88–PS_ and P_12–TT_ were positively correlated with T, Ψ_s_, and water relations. P_12–PS_, P_50–TT_, and P_88–TT_ were negatively correlated with T, Ψ_s_, and water relations. P_50–PS_ was positively correlated with T and Ψ_s_ (Figure 3).

For *R. typhina*, there were significant positive correlations among water relations. P_50–PS_, P_88–PS_, P_50–TT_, and P_88–TT_ were positively correlated with T, Ψ_s_, and water relations. P_12–PS_ and P_12–TT_ were negatively correlated with T, Ψ_s_, and water relations (Figure 4).

**FIGURE 5:**
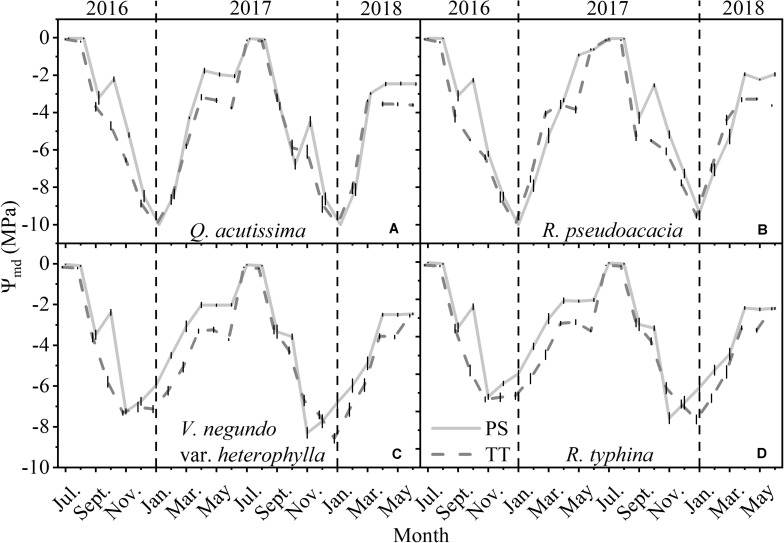
Seasonal changes in midday water potential (Ψ_md_, MPa) of perennial shoots (PS, light gray line) and terminal twigs (TT, gray dash line) throughout 2 years (from July 1st, 2016 to June 30th, 2018). Measurements for *Q*. *acutissima* **(A)**, *R*. *pseudoacacia* **(B)**, *V*. *negundo* var. *heterophylla* **(C)**, and *R*. *typhina* **(D)**. Black dash lines are used to distinguish different years. Data stand for means ± 1 SE, *n* = 15.

The Ψ_md_ of the PS and TT from all species varied monthly and was much larger in summer (June–August) than winter (December–February) (Figure 5). Moreover, the Ψ_md_ for *Q. acutissima* (SD_PS_ = 3.26, SD_TT_ = 3.01) and *R. pseudoacacia* (SD_PS_ = 3.16, SD_TT_ = 2.95) varied considerably more than the Ψ_md_ for *V. negundo* var. *heterophylla* (SD_PS_ = 2.54, SD_TT_ = 2.56) and *R. typhina* (SD_PS_ = 2.50, SD_TT_ = 2.58). During all months the midday water potential differed between PS and TT, with the exception in several winter months including December, January, and February.

**FIGURE 6:**
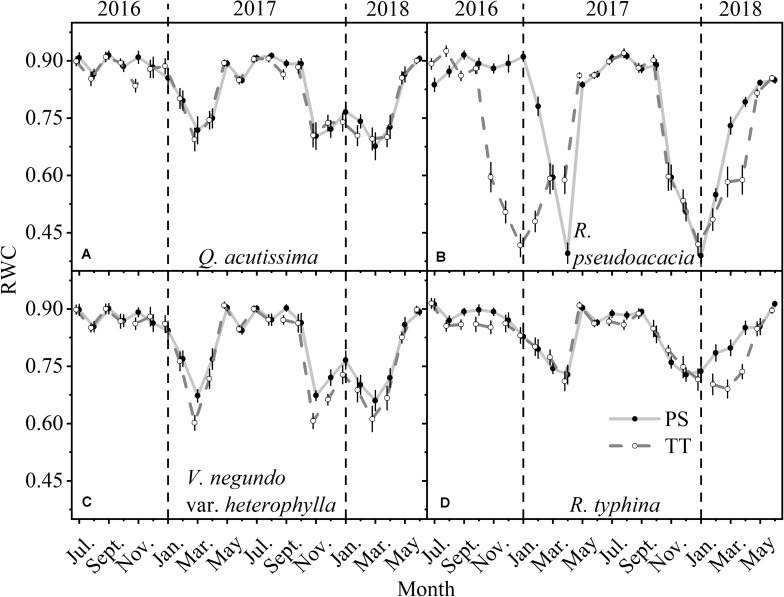
Seasonal changes in relative water content (RWC) of perennial shoots (PS, solid point and light gray solid line) and terminal twigs (TT, hollow point and gray dash line) throughout two successive years (from July 1st, 2016 to June 30th, 2018). Measurements for *Q*. *acutissima* **(A)**, *R*. *pseudoacacia* **(B)**, *V*. *negundo* var. *heterophylla* **(C)**, and *R*. *typhina* **(D)**. Black dash lines are used to distinguish different years. Data stand for means ± 1 SE, *n* = 15.

The RWC of the PS and TT from all species changed monthly and was far higher in summer than winter (Figure 6). Moreover, the RWC for *R. pseudoacacia* (SD_PS_ = 0.16, SD_TT_ = 0.18) varied more than those for *Q. acutissima* (SD_PS_ = 0.08, SD_TT_ = 0.08), *V. negundo* var. *heterophylla* (SD_PS_ = 0.09, SD_TT_ = 0.11), and *R. typhina* (SD_PS_ = 0.06, SD_TT_ = 0.07). The RWC for TT was always lower than that for PS and this difference was highly significant for *R. pseudoacacia*.

**FIGURE 7:**
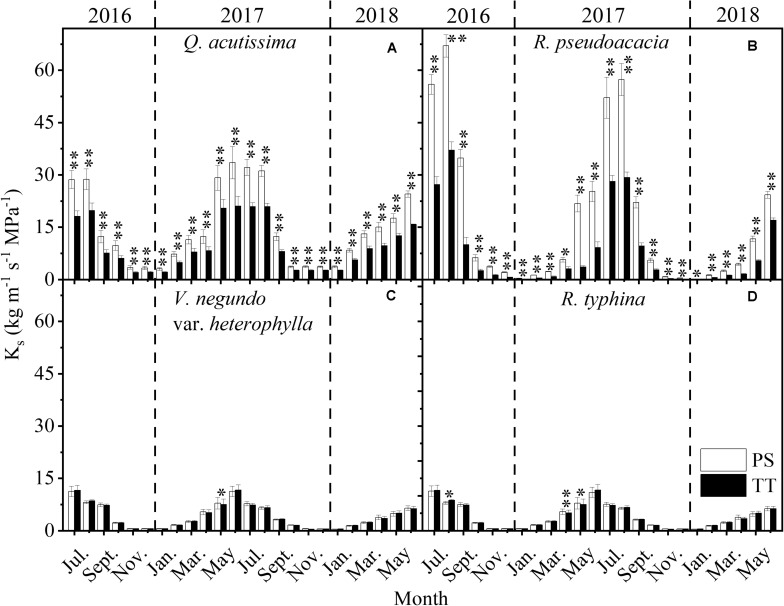
Seasonal changes in stem-specific hydraulic conductivity (K_s_, kg m^–1^ s^–1^ MPa^–1^) of perennial shoots (PS, white column) and terminal twigs (TT, black column) throughout two successive years (from July 1st, 2016 to June 30th, 2018). Measurements for *Q*. *acutissima* **(A)**, *R*. *pseudoacacia* **(B)**, *V*. *negundo* var. *heterophylla* **(C)**, and *R*. *typhina* **(D)**. Dash lines are used to distinguish different years. Data stand for means ± 1 SE, *n* = 15, paired *t*-test was used to detect the differences between PS and TT, *P < 0.05, **P < 0.01.

The K_s_ for the PS and TT from all species varied monthly and was much higher in summer than winter (Figure 7). Moreover, the K_s_ of *R. pseudoacacia* (SD_PS_ = 21.21, SD_TT_ = 11.16) varied substantially more than that for *Q. acutissima* (SD_PS_ = 10.76, SD_TT_ = 7.09) and the K_s_ for the two tree species changed considerably more than those for *V. negundo* var. *heterophylla* (SD_PS_ = 3.47, SD_TT_ = 3.51) and *R. typhina* (SD_PS_ = 3.44, SD_TT_ = 3.52). K_s_ significantly differed between the PS and TT from the two tree species *Q*. *acutissima* and *R*. *pseudoacacia*.

**FIGURE 8:**
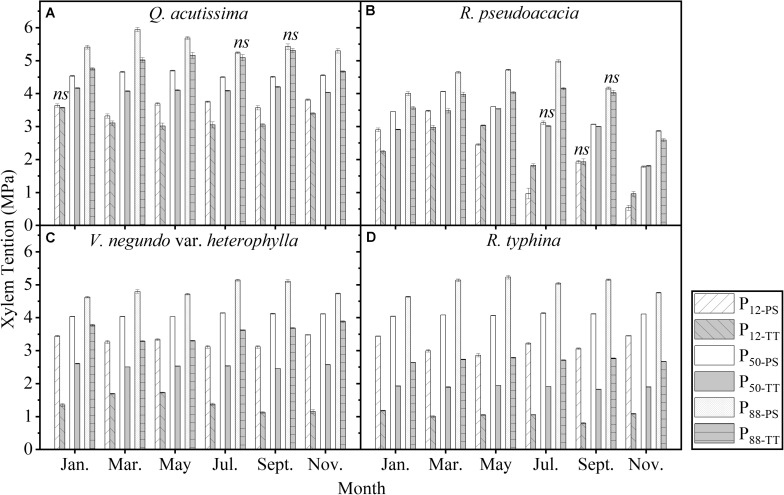
Seasonal changes in embolism resistance traits of perennial shoots (white column) and terminal twigs (light gray column) throughout two successive years (from July 1st, 2016 to June 30th, 2018). Measurements for *Q*. *acutissima* **(A)**, *R*. *pseudoacacia* **(B)**, *V*. *negundo* var. *heterophylla* **(C)**, and *R*. *typhina* **(D)**. P_12–PS_, air-entry point of perennial shoots; P_12–TT_, air-entry point of terminal twigs; P_50–PS_, fastest drop point of perennial shoots; P_50–TT_, fastest drop point of terminal twigs; P_88–PS_, upper inflection point of perennial shoots; P_88–TT_, upper inflection point of terminal twigs. Data stand for means ± 1 SE, *n* = 6. One-tailed paired *t*-test was used to detect the differences, *ns*, no significant difference.

One-tailed paired *t*-test showed that, in nearly all the months and all the species, there were significant differences between PS and TT concerning their embolism resistance traits. The embolism resistance of PS was greater than that of TT (Figure 8).

## Discussion

The results of our study showed that as the seasons changed, the plant hydraulic traits fluctuated. However, the trends of different traits varied, causing tradeoffs or no tradeoffs between traits. Our results indicate that there may be a ubiquitous weak annual tradeoff between plant hydraulic efficiency and safety and strong segmentation among hydraulic traits between PS and TT. The novelty of this research is that the annual variation of plant hydraulic traits is found by monitoring plants for two consecutive years, which is rare in previous studies and provides a prospect for future studies.

### Weak Tradeoff Among Plant Hydraulic Traits

There was substantial consistency between PS and TT concerning their water relations (Figures 1–4). Thus, water relations of the two shrubs were significantly positively correlated with T and Ψ_s_ but not with RH, which may be because the xylem status was conservative to the latter parameter (Jasińska et al., 2015; Alber et al., 2019). In the two shrubs, water relations were significantly correlated, but in the two trees, several traits were not significantly correlated (e.g., RWC and K_s_ of TT in *Q. acutissima*, Ψ_md_ and RWC of PS in *R. pseudoacacia*; Figures 1, 2). One possible explanation is that water resource acquisition is more difficult for trees than shrubs because the former are relatively larger in height and girth (Supplementary Table S1). Thus, their water relations are comparatively independent compared to those of the shrubs. Positive correlations among water relations were weaker for the two trees than the two shrubs as trees are much taller than shrubs (Supplementary Table S1). Trees are more strongly affected by atmospheric demands and soil moisture than shrubs (McDowell and Allen, 2015). Within water relations, no annual tradeoffs were presented, they were coordinated changing with the environmental factors.

The hydraulic efficiency-safety tradeoff consists of negative correlations among hydraulic efficiency (e.g., K_s_) and hydraulic safety (e.g., P_12_, P_50_, P_88_) (Wikberg and Ögren, 2007; Gleason et al., 2016; Petruzzellis et al., 2018; Prendin et al., 2018). Of all the four species, we observed that not all hydraulic safety traits had seasonal tradeoffs with hydraulic efficiency traits. When water relations were conducive to growth, the changes in the embolism resistance traits varied among the four species. Certain embolism resistance traits increased and others decreased regarding safety (Figures 1–4). Although, the xylem structure can acclimate to environmental variation during the growth and development phase of the plant, subsequent acclimation of embolism resistance to environmental stress is not possible because xylem conduits die at maturity (Choat et al., 2012). Several studies claim that plants can alter the concentration of non-structural carbohydrates (NSC) to influence the hydraulic efficiency-safety tradeoff (Bartlett et al., 2014; Delzon, 2015; Wang et al., 2018; Martinez-Vilalta et al., 2019), moreover, seasonal changes in NSC may lead to changes in the hydraulic efficiency-safety tradeoff. The hydraulic safety was not reduced due to the better hydraulic efficiency, but a certain level of embolism resistance could have a tradeoff with the hydraulic efficiency annually, like the tradeoff between K_s_ and P_12_ in *R. pseudoacacia* (Figure 2). Thus, the annual tradeoffs between hydraulic efficiency and safety traits are unclear, several traits may have tradeoffs while others may not, on the whole plant hydraulic traits, the tradeoffs are weak. No consistent results have been reported in previous studies (Liu et al., 2015; Johnson et al., 2016; Blackman et al., 2019; Losso et al., 2019; Skelton et al., 2019). In a global meta-analysis, only ∼1/4 of the studies reviewed supported the safety-efficiency hypothesis while the remaining 3/4 did not (Gleason et al., 2016). According to our results (Figures 1–4), there is a ubiquitous weak annual tradeoff between plant hydraulic efficiency and safety. A ubiquitous weak tradeoff may introduce wide and nearly independent variations in the plant hydraulic traits and make only a limited contribution to species divergence.

### Strong Segmentation Between Perennial Shoots and Terminal Twigs

Hydraulic segmentation reflects the water potential gradient and the difference of hydraulic conductivity between different tissues. Our result showed that for the four species, between PS and TT, the water potential gradient was always there for the whole year (Figure 5) while the difference of hydraulic conductivity existed in only the two tree species (Figure 7). It is more difficult for the two tree species to acquire sufficient water for their branches because of their height and girth (Supplementary Table S1), therefore, they are more likely to adopt a step water supply strategy to protect higher-cost organs from hydraulic failure and maintain hydraulic safety than shrub species (Hartmann et al., 2018; Hammond et al., 2019). Although, RWC is not a key trait of the HSH, it has an inseparable relation with the Ψ_md_ and K_s_ (Martinez-Vilalta et al., 2019). However, there was virtually no obvious annual difference between PS and TT for *Q. acutissima* and the two shrubs. For this reason, their branches had a similar water storage capacity. However, the opposite trend was observed in *R. pseudoacacia* (Figure 6), potentially because *Q. acutissima*, *V. negundo* var. *heterophylla*, and *R. typhina* are more isohydric on the “isohydric-anisohydric” continuous spectrum whereas *R. pseudoacacia* is opposite (Moser et al., 2016; Li et al., 2019). Overall, in the whole year, there is a strong hydraulic segmentation for tree species and a certain degree of hydraulic segmentation for shrub species. The relative differences in hydraulic segmentation between the tree species and the shrub species reflected their divergent responses. This phenomenon might be conducive to niche differentiation and species co-existence.

The vulnerability segmentation of the four species was obvious regarding their embolism resistance traits (Figure 8). All species adopted vulnerability segmentation to cope with variable water availability and seasonal change in the warm temperate zone. Xylem conduits die at maturity. However, as non-structural carbohydrates are fine-tuned (Choat et al., 2012; Bartlett et al., 2014; Delzon, 2015; Wang et al., 2018), synchronous seasonal changes in the embolism resistance traits of PS and TT lay a permanent foundation for hydraulic segmentation. The present study supported the HVSH and corroborated previous reports (Liu et al., 2015; Johnson et al., 2016; Blackman et al., 2019; Losso et al., 2019; Skelton et al., 2019). Strong segmentation among plant hydraulic traits between PS and TT could ensure year-round xylem functionality.

### Diverse Strategies in Different Species

The changes in P_12_ and P_88_ were the opposite between PS and TT (Figures 1–4). Hence, plants actively respond to seasonal changes in embolism resistance and apply a tradeoff strategy (Wikberg and Ögren, 2007). Different species implement various hydraulic efficiency-safety tradeoffs to adapt to seasonal changes in the warm temperate zone (Wanploy et al., 2015; Aguilar-Romero et al., 2017; Santiago et al., 2018). One strategy is conservative (high hydraulic safety, low hydraulic efficiency). In spring and summer, P_12_ decreases, P_50_ and P_88_ increase. Plants exhibit a moderate hydraulic efficiency and safety tradeoff strategy, which shows that plants adapt to the changeable water conditions in the warm temperate zone (Luan et al., 2011; Corlett, 2016). In autumn and winter, P_12_ increases, P_50_ and P_88_ decrease. Rapid air diffusion and embolism occur, and the plants prepare for dormancy. The other strategy is risky (high hydraulic efficiency, low hydraulic safety). In spring and summer, P_12_ increases, P_50_ and P_88_ decrease. Plants take a high hydraulic efficiency and low hydraulic safety tradeoff strategy, which makes plants grow fast but is not conducive to plants adapting to the changeable water conditions in the warm temperate zone. In autumn and winter, P_12_ decreases, P_50_ and P_88_ increase. The air penetrates easily but does not readily diffuse. This phenomenon partially prolongs plant growth but the residual water might increase the risk of freeze-thaw embolism (Niu et al., 2017; Yin et al., 2018). For *Q. acutissima* (Figure 1), a conservative TT strategy combined with a risky PS strategy ensured hydraulic safety and kept K_s_ at a high level. For *R. pseudoacacia* (Figure 2), a risky strategy for all branches furnished sufficient water under favorable conditions. However, the hydraulics were not safe and embolism could have occurred under poor water conditions. For *V. negundo* var. *heterophylla* (Figure 3), a risky TT strategy combined with a conservative PS strategy accommodated rapid branch growth under favorable water conditions but supplied the older branches when the water conditions were poor. For *R. typhina* (Figure 4), conservative strategies for both PS and TT ensured hydraulic safety for all branches. In general, on the “isohydric-anisohydric” ontinuous spectrum, anisohydric species assumed a less safe, more efficient strategy than isohydric species (Moser et al., 2016; Aguilar-Romero et al., 2017; Petruzzellis et al., 2018; Santiago et al., 2018; Li et al., 2019). Species may assume diverse strategies to adapt to changing environments. Those adopting a risky strategy could spread to drier areas to increase their chances of survival while those adopting a conservative strategy might propagate only locally. In this case, habitat partitioning and niche divergence of sympatric species will increase (Aguilar-Romero et al., 2017; Santiago et al., 2018; Rosas et al., 2019).

## Conclusion

The present study disclosed that *R. pseudoacacia* implements a risky strategy while *R. typhina* adopts a conservative strategy. In contrast, the other two species assume both strategies in an attempt to adapt to environmental changes. They maintain xylem functionality via a weak efficiency-safety tradeoff of hydraulic traits and strong annual segmentation between perennial shoots and terminal twigs. Our research reveals annual weak tradeoff and strong segmentation of hydraulic traits and confirms that various species differ concerning their hydraulic strategies. Thus, habitat partitioning and niche divergence under a changing environment are created in the warm temperate zone.

## Data Availability Statement

The original contributions presented in the study are included in the article/Supplementary Material, further inquiries can be directed to the corresponding author.

## Author Contributions

XL designed the study, conducted field and laboratory measurements, and analyzed the data. ND, HW, and RW designed the study and secured funding. QL, FW, and XS contributed to laboratory measurements and data analysis. NW, HS, RC, and PW conducted data analysis. XL wrote the manuscript that was intensively edited by all authors.

## Conflict of Interest

The authors declare that the research was conducted in the absence of any commercial or financial relationships that could be construed as a potential conflict of interest.

## Supplementary Material

Supplementary Figure 1 Environmental factors including **(A)** temperature (T, °C), **(B)** monthly average precipitation (MAP, mm), **(C)** relative humidity (RH), and **(D)** soil water potential (Ψ_s_, MPa) from July 2016 to June 2018. In **(D)**, the solid lines represent the average soil water potential.

Supplementary Table 1 Basic information of *Q. acutissima*, *R. pseudoacacia*, *V. negundo* var. *heterophylla*, *R. typhina* L. Data stand for mean SD, *n* = 15.

## Abbreviations

BD: basal diameter
CDF: cumulative distribution function
DBH: diameter at breast height
HSH: hydraulic segmentation hypothesis
HVSH: hydraulic vulnerability segmentation hypothesis
K_h_: hydraulic conductivity
K_m_: maximum hydraulic conductivity
K_s_: stem-specific hydraulic conductivity
K_s–PS_: stem-specific hydraulic conductivity of perennial shoots
K_s–TT_: stem-specific hydraulic conductivity of terminal twigs
NSC: non-structural carbohydrates
PLC: percentage loss of hydraulic conductivity
MAP: monthly average precipitation
P: the progressively increased air-injection pressure
P_12_: air-entry point
P_12–PS_: air-entry point of perennial shoots
P_12–TT_: air-entry point of terminal twigs
P_50_: fastest drop point
P_50–PS_: fastest drop point of perennial shoots
P_50–TT_: fastest drop point of terminal twigs
P_88_: upper inflection point
P_88–PS_: upper inflection point of perennial shoots
P_88–TT_: upper inflection point of terminal twigs
PS: perennial shoots
Q_m_: mass of water through stem per unit time
RWC: relative water content
RWC_PS_: relative water content of perennial shoots
RWC_TT_: relative water content of terminal twigs
SD: standard deviation
SE: standard error
TT: terminal twigs
W_d_: dry weight
W_f_: fresh weight
W_s_: saturated weight
Ψ_md_: midday water potential
Ψ_md–PS_: midday water potential of perennial shoots
Ψ_md–TT_: midday water potential of terminal twigs
Ψ_pd_: predawn water potential
Ψ_s_: soil water potential.

## References

[B1] Aguilar-Romero R. F. Pineda-Garcia H. Paz A. González-Rodríguez A. Oyama K. (2017). Differentiation in the water-use strategies among oak species from central Mexico. *Tree Physiol.* 37 915–925. 10.1093/treephys/tpx033 28369608

[B2] Alber M. Petit G. Sellin A. (2019). Does elevated air humidity modify hydraulically relevant anatomical traits of wood in Betula pendula? *Trees* 33 1361–1371. 10.1007/s00468-019-01863-0

[B3] Allen C. D. Macalady A. K. Chenchouni H. Bachelet D. McDowell N. Vennetier M. (2010). A global overview of drought and heat-induced tree mortality reveals emerging climate change risks for forests. *For. Ecol. Manag.* 259 660–684. 10.1016/j.foreco.2009.09.001

[B4] Aranda I. Gil L. Pardos J. A. (2005). Seasonal changes in apparent hydraulic conductance and their implications for water use of European beech (*Fagus sylvatica* L.) and sessile oak [*Quercus petraea* (Matt.) Liebl] in South Europe. *Plant Ecol.* 179 155–167. 10.1007/s11258-004-7007-1

[B5] Bartlett M. K. Zhang Y. Kreidler N. Sun S. Ardy R. Cao K. (2014). Global analysis of plasticity in turgor loss point, a key drought tolerance trait. *Ecol. Lett.* 17 1580–1590. 10.1111/ele.12374 25327976

[B6] Blackman C. J. Li X. Choat B. Rymer P. D. De Kauwe M. G. Duursma R. A. (2019). Desiccation time during drought is highly predictable across species of Eucalyptus from contrasting climates. *New Phytol.* 224 632–643. 10.1111/nph.16042 31264226

[B7] Charrier G. S. Delzon J. C. Domec L. Zhang L. Delmas C. Merlin I. (2018). Drought will not leave your glass empty: low risk of hydraulic failure revealed by long-term drought observations in world’s top wine regions. *Sci. Adv.* 4:eaao6969. 10.1126/sciadv.aao6969 29404405 PMC5796794

[B8] Choat B. Brodribb T. J. Brodersen C. R. Duursma R. A. López R. Medlyn B. E. (2018). Triggers of tree mortality under drought. *Nature* 558 531–539. 10.1038/s41586-018-0240-x 29950621

[B9] Choat B. Jansen S. Brodribb T. J. Cochard H. Delzon S. Bhaskar R. (2012). Global convergence in the vulnerability of forests to drought. *Nature* 491 752–755. 10.1038/nature11688 23172141

[B10] Corlett R. T. (2016). The impacts of droughts in tropical forests. *Trends Plant Sci.* 21 584–593. 10.1016/j.tplants.2016.02.003 26994658

[B11] Del Grosso S. Parton W. Stohlgren T. Zheng D. Bachelet D. Prince S. (2008). Global potential net primary production predicted from vegetation class, precipitation, and temperature. *Ecology* 89 2117–2126. 10.1890/07-0850.1 18724722

[B12] Delzon S. (2015). New insight into leaf drought tolerance. *Funct. Ecol.* 29 1247–1249. 10.1111/1365-2435.12500

[B13] Domec J. C. Gartner B. L. (2001). Cavitation and water storage capacity in bole xylem segments of mature and young Douglas-fir trees. *Trees* 15 204–214. 10.1007/s004680100095

[B14] Du N. Tan X. Li Q. Liu X. Zhang W. Wang R. (2017). Dominance of an alien shrub Rhus typhina over a native shrub *Vitex negundo* var. heterophylla under variable water supply patterns. *PLoS One* 12:e0176491. 10.1371/journal.pone.0176491 28445505 PMC5406003

[B15] Easterling D. R. Meehl G. A. Parmesan C. Changnon C. A. Karl T. R. Mearns L. O. (2000). Climate extremes: observations, modeling, and impacts. *Science* 289 2068–2074. 10.1126/science.289.5487.2068 11000103

[B16] Esquivel-Muelbert A. Baker T. R. Dexter K. G. Lewis S. L. Steege H. Lopez-Gonzalez G. (2017). Seasonal drought limits tree species across the Neotropics. *Ecography* 40 618–629. 10.1111/ecog.01904

[B17] Fang J. Wang Z. Tang Z. (2011). *Atlas of Woody Plants in China.* Beijing: Higher Education Press.

[B18] Ge C. Yu X. Kan M. Qu C. (2017). Adaption of *Ulva pertusa* to multiple-contamination of heavy metals and nutrients: biological mechanism of outbreak of *Ulva* sp. green tide. *Mar, Pollut. Bull.* 125 250–253. 10.1016/j.marpolbul.2017.08.025 28826924

[B19] Gimbel K. F. Felsmann K. Baudis M. Puhlmann H. Gessler A. Bruelheide H. (2015). Drought in forest understory ecosystems – a novel rainfall reduction experiment. *Biogeosciences* 12 961–975. 10.5194/bg-12-961-2015

[B20] Gleason S. M. Westoby M. Jansen S. Choat B. Hacke U. G. Pratt R. B. (2016). Weak tradeoff between xylem safety and xylem-specific hydraulic efficiency across the world’s woody plant species. *New Phytol.* 209 123–136. 10.1111/nph.13646 26378984

[B21] Grossiord C. Ulrich D. E. M. Vilagrosa A. (2020). Controls of the hydraulic safety–efficiency trade-off. *Tree Physiol.* 40:taa013. 10.1093/treephys/tpaa013 32050013

[B22] Hammond W. M. Yu K. Wilson L. A. Will R. E. Anderegg W. R. L. Adams H. D. (2019). Dead or dying? Quantifying the point of no return from hydraulic failure in drought-induced tree mortality. *New Phytol.* 223 1834–1843. 10.1111/nph.15922 31087656 PMC6771894

[B23] Hartmann H. Adams H. D. Hammond W. M. Hoch G. Landhäusser S. M. Wiley E. (2018). Identifying differences in carbohydrate dynamics of seedlings and mature trees to improve carbon allocation in models for trees and forests. *Environ. Exp. Bot.* 152 7–18. 10.1016/j.envexpbot.2018.03.011

[B24] Högy P. Poll C. Marhan S. Kandeler E. Fangmeier A. J. F. C. (2013). Impacts of temperature increase and change in precipitation pattern on crop yield and yield quality of barley. *Food Chem.* 136 1470–1477. 10.1016/j.foodchem.2012.09.056 23194550

[B25] Holmlund H. I. Lekson V. M. Gillespie B. M. Nakamatsu N. A. Burns A. M. Sauer K. E. (2016). Seasonal changes in tissue-water relations for eight species of ferns during historic drought in California. *Am. J. Bot.* 103 1607–1617. 10.3732/ajb.1600167 27638918

[B26] Jaquish L. L. Ewers F. W. (2001). Seasonal conductivity and embolism in the roots and stems of two clonal ring-porous trees, *Sassafras albidum* (*Lauraceae*) and *Rhus typhina* (*Anacardiaceae*). *Am. J. Bot.* 88 206–212. 10.2307/2657011 11222243

[B27] Jasińska A. Alber M. Tullus A. Rahi M. Sellin A. (2015). Impact of elevated atmospheric humidity on anatomical and hydraulic traits of xylem in hybrid aspen. *Funct. Plant Biol.* 42 565–578. 10.1071/FP14224 32480701

[B28] Jin Y. Wang C. Zhou Z. (2018). Conifers but not angiosperms exhibit vulnerability segmentation between leaves and branches in a temperate forest. *Tree Physiol.* 39 454–462. 10.1093/treephys/tpy111 30321431

[B29] Johnson D. M. Wortemann R. McCulloh K. A. Jordan-Meille L. Ward W. Warren J. M. (2016). A test of the hydraulic vulnerability segmentation hypothesis in angiosperm and conifer tree species. *Tree Physiol.* 36 983–993. 10.1093/treephys/tpw031 27146334

[B30] Levionnois S. Ziegler C. Jansen S. Calvet E. Coste S. Stahl C. (2020). Vulnerability and hydraulic segmentations at the stem–leaf transition: coordination across Neotropical trees. *New Phytol.* 228 512–524. 10.1111/nph.16723 32496575

[B31] Li M. Guo W. Du N. Xu Z. Guo X. (2018). Nitrogen deposition does not affect the impact of shade on *Quercus acutissima* seedlings. *PLoS One* 13:e0194261. 10.1371/journal.pone.0194261 29534093 PMC5849318

[B32] Li Q. Wang N. Liu X. Liu S. Wang H. Zhang W. (2019). Growth and physiological responses to successional water deficit and recovery in four warm-temperate woody species. *Physiol. Plant.* 167 645–660. 10.1111/ppl.12922 30637759

[B33] Liu Y.-Y. Song J. Wang M. Li N. Niu C.-Y. Hao G.-Y. (2015). Coordination of xylem hydraulics and stomatal regulation in keeping the integrity of xylem water transport in shoots of two compound-leaved tree species. *Tree Physiol.* 35 1333–1342. 10.1093/treephys/tpv061 26209618

[B34] Losso A. Bär A. Dämon B. Dullin C. Ganthaler A. Petruzzellis F. (2019). Insights from in vivo micro-CT analysis: testing the hydraulic vulnerability segmentation in *Acer pseudoplatanus* and *Fagus sylvatica* seedlings. *New Phytol.* 221 1831–1842. 10.1111/nph.15549 30347122 PMC6492020

[B35] Luan J. Liu S. Wang J. Zhu X. Shi Z. (2011). Rhizospheric and heterotrophic respiration of a warm-temperate oak chronosequence in China. *Soil Biol. Biochem.* 43 503–512. 10.1016/j.soilbio.2010.11.010

[B36] Magnani F. Borghetti M. (1995). Interpretation of seasonal changes of xylem embolism and plant hydraulic resistance in *Fagus sylvatica*. *Plant Cell Environ.* 18 689–696. 10.1111/j.1365-3040.1995.tb00570.x

[B37] Martinez-Vilalta J. Anderegg W. R. L. Sapes G. Sala A. (2019). Greater focus on water pools may improve our ability to understand and anticipate drought-induced mortality in plants. *New Phytol.* 223 22–32. 10.1111/nph.15644 30560995

[B38] McDowell N. Allen C. (2015). Darcy’s law predicts widespread forest mortality under climate warming. *Nat. Clim Change* 5 669–672. 10.1038/nclimate2641

[B39] Moser A. Rötzer T. Pauleit S. Pretzsch H. (2016). The urban environment can modify drought stress of small-leaved lime (*Tilia cordata* Mill.) and black locust (*Robinia pseudoacacia* L.). *Forests* 7:71 10.3390/f7030071

[B40] Ni J. (2003). Plant functional types and climate along a precipitation gradient in temperate grasslands, north-east China and south-east Mongolia. *J. Arid Environ.* 53 501–516. 10.1006/jare.2002.1063

[B41] Niu C.-Y. Meinzer F. C. Hao G.-Y. (2017). Divergence in strategies for coping with winter embolism among co-occurring temperate tree species: the role of positive xylem pressure, wood type and tree stature. *Funct. Ecol.* 31 1550–1560. 10.1111/1365-2435.12868

[B42] Pammenter N. W. Van der Willigen C. (1998). A mathematical and statistical analysis of the curves illustrating vulnerability of xylem to cavitation. *Tree Physiol.* 18 589–593. 10.1093/treephys/18.8-9.589 12651346

[B43] Pérez-Harguindeguy N. Díaz S. Garnier E. Lavorel S. Poorter H. Jaureguiberry H. (2016). New handbook for standardised measurement of plant functional traits worldwide. *Aust. J. Bot.* 61 167–234. 10.1071/BT12225

[B44] Petruzzellis F. Nardini A. Savi T. Tonet V. Castello M. Bacaro G. (2018). Less safety for more efficiency: water relations and hydraulics of the invasive tree *Ailanthus altissima* (Mill.) Swingle compared with native *Fraxinus ornus* L. *Tree Physiol.* 39 76–87. 10.1093/treephys/tpy076 29982793

[B45] Prendin A. L. Mayr S. Beikircher B. von Arx G. Petit G. (2018). Xylem anatomical adjustments prioritize hydraulic efficiency over safety as Norway spruce trees grow taller. *Tree Physiol.* 38 1088–1097. 10.1093/treephys/tpy065 29920598

[B46] Rosas T. Mencuccini M. Barba J. Cochard H. Saura-Mas S. Martínez-Vilalta J. (2019). Adjustments and coordination of hydraulic, leaf and stem traits along a water availability gradient. *New Phytol.* 223 505–507. 10.1111/nph.15684 30636323

[B47] Santiago L. S. De Guzman M. E. Baraloto C. Vogenberg J. E. Brodie M. Hérault B. (2018). Coordination and trade-offs among hydraulic safety, efficiency and drought avoidance traits in Amazonian rainforest canopy tree species. *New Phytol.* 218 1015–1024. 10.1111/nph.15058 29457226

[B48] Scholz F. G. Bucci S. J. Goldstein G. (2014). Strong hydraulic segmentation and leaf senescence due to dehydration may trigger die-back in Nothofagus dombeyi under severe droughts: a comparison with the co-occurring *Austrocedrus chilensis*. *Trees* 28 1475–1487. 10.1007/s00468-014-1050-x

[B49] Skelton R. P. Anderegg L. D. L. Papper P. Reich E. Dawson T. E. Kling M. (2019). No local adaptation in leaf or stem xylem vulnerability to embolism, but consistent vulnerability segmentation in a North American oak. *New Phytol.* 223 1296–1306. 10.1111/nph.15886 31059125

[B50] Sperry J. S. Taneda H. Bush S. E. Hacke U. G. (2008). Evaluation of centrifugal methods for measuring xylem cavitation in conifers, diffuse and ringporous angiosperms. *New Phytol.* 177 558–568. 10.1111/j.1469-8137.2007.02272.x 18028295

[B51] Torres-Ruiz J. M. Cochard H. Choat B. Jansen S. López R. Tomášková I. (2017). Xylem resistance to embolism: presenting a simple diagnostic test for the open vessel artefact. *New Phytol.* 215 489–499. 10.1111/nph.14589 28467616

[B52] van der Sande M. T. Poorter L. Schnitzer S. A. Engelbrecht B. M. J. Markesteijn L. (2019). The hydraulic efficiency–safety trade-off differs between lianas and trees. *Ecology* 100:e02666. 10.1002/ecy.2666 30801680 PMC6850011

[B53] Varela M. C. Reinoso H. Luna V. Cenzano A. M. (2018). Seasonal changes in morphophysiological traits of two native Patagonian shrubs from Argentina with different drought resistance strategies. *Plant Physiol. Biochem.* 127 506–515. 10.1016/j.plaphy.2018.03.018 29709880

[B54] Wang A.-Y. Han S.-J. Zhang J.-H. Wang M. Yin X.-H. Fang L.-D. (2018). The interaction between nonstructural carbohydrate reserves and xylem hydraulics in Korean pine trees across an altitudinal gradient. *Tree Physiol.* 38 1792–1804. 10.1093/treephys/tpy119 30376119

[B55] Wang R. Zhou G. (2000). *The Vegetation of Shandong Province.* Qingdao: Shandong University of Science and Technology.

[B56] Wanploy J. Ratchanee R. Krissada S. Têtè Sévérien B. Frederic G. Hervé C. (2015). Clonal variability for vulnerability to cavitation and other drought-related traits in *Hevea brasiliensis* Müll. *J. Plant Hydraul.* 2:e001 10.20870/jph.2015.e001

[B57] Wason J. W. Anstreicher K. S. Stephansky N. Huggett B. A. Brodersen C. R. (2018). Hydraulic safety margins and air-seeding thresholds in roots, trunks, branches and petioles of four northern hardwood trees. *New Phytol.* 219 77–88. 10.1111/nph.15135 29663388

[B58] Wikberg J. Ögren E. (2007). Variation in drought resistance, drought acclimation and water conservation in four willow cultivars used for biomass production. *Tree Physiol.* 27 1339–1346. 10.1093/treephys/27.9.1339 17545133

[B59] Xu F. Guo W. Wang R. Xu W. Du N. Wang Y. (2009). Leaf movement and photosynthetic plasticity of black locust (*Robinia pseudoacacia*) alleviate stress under different light and water conditions. *Acta Physiol. Plant.* 31 553–563. 10.1007/s11738-008-0265-0

[B60] Yin X.-H. Sterck F. Hao G.-Y. (2018). Divergent hydraulic strategies to cope with freezing in co-occurring temperate tree species with special reference to root and stem pressure generation. *New Phytol.* 219 530–541. 10.1111/nph.15170 29682759

[B61] Zhang W. Feng F. Tyree M. T. (2018). Seasonality of cavitation and frost fatigue in Acer mono Maxim. *Plant Cell Environ.* 41 1278–1286. 10.1111/pce.13117 29220549

