# Supplementary material for: Weak Tradeoff and Strong Segmentation Among Plant Hydraulic Traits During Seasonal Variation in Four Woody Species
Source: Front Plant Sci. 2020 Nov 24;11:585674. doi: 10.3389/fpls.2020.585674 (PMC7732674; doi:10.3389/fpls.2020.585674)
Supplement: Supplementary Table 1 — Basic information of Q. acutissima, R. pseudoacacia, V. negundo var. heterophylla, R. typhina L. Data stand for mean SD, n = 15. [file Table_1.DOCX]

**Table S1** Basic information of *Q. acutissima*, *R. pseudoacacia*, *V. negundo* var. *heterophylla*, *R. typhina* L. Data stand for mean ± SD, *n* = 15.

| Species | *Q. acutissima* | *R. pseudoacacia* | *V. negundo* var. *heterophylla* | *R. typhina* |
| --- | --- | --- | --- | --- |
| Family | Fagaceae | Fabaceae | Lamiaceae | Anacardiaceae |
| Genus | *Quercus* | *Robinia* | *Vitex* | *Rhus* |
| Life form | tree | tree | shrub | shrub |
| Leaf form | simple leaf | compound leaf | compound leaf | compound leaf |
| Origin | native | alien | native | alien |
| Age | 10 | 10 | 10 | 10 |
| H (m) | 9.50 ± 0.11 | 8.03 ± 0.10 | 2.83 ± 0.12 | 3.50 ± 0.11 |
| DBH (cm) | 23.57 ± 0.30 | 23.43 ± 0.35 | — | — |
| BD (cm) | — | — | 3.24 ± 0.09 | 5.31 ± 0.13 |

*Note.* H, height, m; DBH, diameter at breast height, cm; BD, basal diameter, cm.
